# The relationship between gender and cultural beliefs of malaria into typhoid progression among rural rice farmers in Central Kenya

**DOI:** 10.4314/ahs.v24i3.13

**Published:** 2024-09

**Authors:** Dawit Woldu

**Affiliations:** University of Houston Clear Lake College of Human Sciences and Humanities, Anthropology and Cross-cultural and Global Studies

**Keywords:** Malaria, Typhoid, illness progression, cultural beliefs, gender disparity in Health

## Abstract

**Background:**

Women have an increased risk of contracting malaria in Kenya and the developing world because of gender roles and the cultural belief systems about disease progression and treatment. Cultural belief systems about illness progression have important implications for gender-based health intervention.

**Objectives:**

The main objective of this research is to explore how rice farming Kikuyu ethnic group in the Mwea division of central Kenya cultural beliefs about malaria into typhoid progression. It also aims to examine the association between gender and malaria into typhoid progression cultural belief system.

**Design:**

The study employs cross-sectional study design (N=250). Variables related to demographic and cultural beliefs on malaria into typhoid progression were collected using a structured questionnaire.

**Results:**

More than 62% of women and 47% of men interviewed adhere to malaria into typhoid progression belief system. Multivariable logistic analysis shows women are more than twice as likely than men to believe in malaria into typhoid progression (aOR 2.15; 95% CI 1.21, 3.79, p < 0.009).

**Conclusion:**

The study demonstrates the underlying cultural factors related to the gender disparity in the appropriate knowledge on cultural belief system of malaria into typhoid progression which could influence the overall women's health outcomes in developing countries.

## Introduction

Malaria continues to be the major cause of morbidity and mortality in many sub-Saharan African countries. Despite remarkable progress against malaria globally, Africa accounts for 90% of malaria cases and 92% of malaria deaths as recently as 2015.^1^,^2^ Women and children are at greater risk of contracting malaria than other demographic groups.^3^ Access to anti-malaria drugs and prevention continues to be poor in the region with 69% of pregnant women not receiving 3 doses of IPTp, an intermittent preventive malaria treatment drug.^1^ According to the Presidential Malaria Initiative 2016 report, malaria remains a major health problem in Kenya with 80% of the population at risk of contracting the disease. Infectious pattern modelling has shown an association between malaria and typhoid which provides a robust co-existence of the two illnesses in vulnerable communities in Africa.^4^,^5^ Agricultural communities, like the one in Mwea, are especially at a greater risk of malaria than other economic sectors because they live in a mosquito prone environment. The gendered nature of rice and other agricultural farming in Kenya and the region put women at greater malaria risk than their men counter parts^3^. Many communities in sub-Saharan Africa are forced to use local and culturally based resources to deal with the threat of malaria. Home-based treatment and the use of herbal-based traditional healers are the primary care providers in many remote villages.^6^-^9^

This study adopts a cultural explanatory models of illness theoretical framework.^10^,^11^ This theoretical framework assumes there is culturally embedded ways of identifying and experiencing illness process.^10^ It provides a framework how lay people within a specific culture group understand and explain illness causes, symptoms and treatment.^10^,^11^ Different cultures have unique perceptions about the disease prognosis, causes, and treatment. Theunderstanding of how people perceive and diagnose malaria becomes even more important in societies where self-treatment of illness is common. Recognition of the illness, definition, and management are situated in a socio-cultural context and reflect social class, age, gender, and level of education.^7^,^12^,^13^ The gender factors play both at the cultural perception and diagnosis of the illness^7^,^14^ and the negative consequences of these cultural perceptions.^3^,^6^ Over the years, anthropologists have identified many potential challenges for clinicians and epidemiologists because the indigenous etiology of the illness might not be compatible with the clinical and epidemiological definition of malaria. Many cultures in the African continent overlap malaria symptoms with other infectious illnesses varying by gender and age.^15^-^18^

Public health and Anthropological studies from the developing world underline the importance of indigenous cultural construction of illness and illness experience. Previous socio-cultural studies of malaria, in Africa, focused on the local understanding of the disease severity and cross-cultural understanding of malaria. Specifically, these studies focused on how societies understand the causes, diagnosis, and treatment of malaria along gender and age. For example, mothers in the coastal regions of Kenya assign three stages of malaria symptoms in children into mundane, mild, and severe.^7^ However, this malaria recognition process does not apply to adults. Similarly, studies by Kamat^14^ and Langwick^19^ in Tanzania showed that Degedege, a clinical malaria but locally considered a different illness, only impacts children. Some cultures may classify clinical malaria symptoms into several distinct illnesses. Most often laypeople in several cultures in different cultures in Africa overlap malaria symptoms with pneumonia, typhoid, and some diarrheal diseases.^20^,^21^ In this case they name the illness as malaria or typhoid depending on the salient symptom combination that defines a specific illness. In some cultures, malaria is lumped into a larger illness category.^15^,^22^ While understanding of the cultural construction of the disease causes, recognition, and treatment is very important, our cultural understanding of the progression of one illness into a different illness along gender lines is poorly understood. There is very limited research both in Kenya and other African cultures that shows the progression of malaria into other forms of diseases and the gender dynamics associated with it.

This study draws a quantitative approach to examining the indigenous beliefs of malaria etiology to illustrate how indigenous cultural understanding of malaria to typhoid progression is constructed among the Kikuyu of central Kenya along gender lines. Communities in Mwea understand malaria and typhoid as an illness in a continuum of severity rather than separate illnesses with different causes and symptoms.^6^ The study hypothesizes there is a gender difference about the cultural understanding of malaria to typhoid progression.

## Materials and methods

**Setting:** The study is based on a 13 month-long study of a cross-sectional survey study. I conducted this research in collaboration with the Kenyan Medical Research Institute (KEMRI). This research was conducted in Mwea Division of Central Kenya about 100km northeast of Nairobi. The research site lays on the swampy rice fields at the bottom of Mount Kenya. Malaria is endemic and the number one health problem in this division.^3^ Mwea has a conducive climatic conditions for the breeding of malaria vectors, with an annual rain fall range of 1200-1600mm per year with some seasonal variability.^23^ The site is located at about 1162 meters above sea level with annual mean temperature of 19.7-23.9 degree Celsius.^24^ Mwea represents the economic and cultural hub of the surrounding rural communities with rapid urbanization and economic expansion. Much of the economic growth and urbanization is driven by government and community sector supported rice irrigation. About 90% of Kenyan rice supplies comes from this division. It is a relatively culturally diverse but heavily inhabited by Kikuyu speakers. Despite the presence of modern hospitals and private pharmacies, most people in rural areas self-treat infections and other illness.^3^

**Research Design:** The study uses a cross-sectional non-randomized study. The data collection took place in two phases over a 13-month period between 2010-2013. A cross-sectional study was conducted to understand the level of community congruency to the malaria to typhoid progression cultural belief system. More specifically, its main objective was to explore the association between the different exposure variables with the outcome variable of interest. Furthermore, the objective was to examine the variables that best predict malaria to typhoid progression.

This cross-sectional survey employed a purposive sampling with 250 informants, with an equal number of men and women from three non-irrigated villages, four irrigated villages, and the Mwea town. The survey questionnaire collected data on socio-cultural, ecological, and cultural health beliefs.

Compliance with Ethnical Standards: Interview questionnaires and consent forms were translated to local languages (Kiswahili and Kikuyu) by a local language expert. All participants were briefed on the goal and purpose of the study and were asked to participate in the study voluntarily. Informed consent was obtained from all individuals included in the study. No names or other identifying information were included in the questionnaire and participants were guaranteed confidentiality of information. Interviews were collected in a secured place ithin private residences or workplaces. Most of the interviews were conducted in Swahili and Kikuyu with a few in English. Ethical approval for the study was obtained from the author's University Institutional Review Board (IRB) and KEMRI's Scientific Steering Committee and Ethical Review Committee.

### Variables

#### Dependent Variables

The dependent variable was a question that assessed an individual's beliefs about malaria to typhoid progression. Based on the responses, the variable was dichotomized as “Yes” and “No”; “Yes” implying individuals' belief in malaria to typhoid progression and “No” indicating individuals do not believe in malaria to typhoid progression.

#### Independent variables

The study assessed several exposures including age (<30, 30-55, >55), gender (female, male), level of education (no education, primary education, secondary education or more), belief in non-mosquito causes (yes or no), bednet use (all the time, sometimes, never); and distance to the hospital (>1 hour, 2 hours > 2 hours), and Self ascribed Socio-economic Status (SES) (high/middle and low income). The study employed socially, and locally meaningful SES measurement based on previous studies, in which respondents were asked to which of the locally used SES category they belong to.^3^,^6^

### Analysis

The survey data was assessed using SAS 9.4 (SAS Institute, Inc., Cary, NC). Descriptive statistics were used to summarize and describe the distribution of different variables. Using chi-square test statistic, bivariate analysis was performed to determine association between outcome and all the exposure variables. Univariate and multivariate logistic regression analysis was performed to determine exposures that were significantly associated with the outcome of interest. To control for confounding, all variables were retained in the multivariable logistic regression regardless of statistical significance in the bivariate analysis. Odds ratio (OR), 95% confidence interval (95% CI), and p-value were determined for each of the independent variables. P value < 0.05 was considered statistically significant. Potential multicollinearity was assessed using variance inflation factor and tolerance. Results revealed that the regression analysis was not prone to multicollinearity.

## Results

The purposive analytical sample included 250 study participants. Overall, 49.6% study participants were 31-55 years old, 50.4% were male, 51.2% had primary level education, 57.3% belonged to middle/high income category, 16.1% lived in urban areas, 40.3% did not possess any land, 3.2% did not use bed net, 55.2% believed the etiology of malaria was not mosquito related, and 54.8% believed in malaria to typhoid progression (Table 1). The analysis shows 62.7 % of females and 47.2% males believe malaria to typhoid progression. In ,the bivariate analysis gender and bed net use were significantly associated with the belief in malaria to typhoid transmission (Table 1).

**Table 1:** Characteristics of the study sample by Malaria to Typhoid progression (n=248)

	Overall	Malaria to typhoid		

		No	Yes	
	n (%)	n (%)	n (%)	*p* value
**Age**				0.872
≤30 years	71 (28.6)	32 (45.1)	39 (54.9)	
31 - 55 years	123 (49.6)	54 (43.9)	69 (56.1)	
>55 years	54 (21.8)	26 (48.2)	28 (51.8)	
**Gender**				0.015
Female	123 (49.6)	46 (37.4)	77 (62.6)	
Male	125 (50.4)	66 (52.8)	59 (47.2)	
**Education**				0.404
None	25 (10.1)	15 (60.0)	10 (40.0)	
Primary	127 (51.2)	53 (41.7)	74 (58.3)	
Secondary	86 (34.7)	39 (45.4)	47 (54.6)	
Tertiary	10 (4.0)	5 (50.0)	5 (50.0)	
**Self-ascribed SES**				0.209
Low income	106 (42.7)	43 (40.6)	63 (59.4)	
Middle/high income	142 (57.3)	69 (48.6)	73 (51.4)	
**Residence**				0.322
Non irrigated	89 (35.9)	35 (39.3)	54 (60.7)	
Irrigated	119 (48.0)	56 (47.1)	63 (52.9)	
Urban	40 (16.1)	21 (52.5)	19 (47.5)	
**Land in hectars**				0.581
No land	100 (40.3)	44 (44.0)	56 (56.0)	
1-2 hectares	87 (35.1)	37 (42.5)	50 (57.5)	
>2 hectares	61 (24.6)	31 (50.8)	30 (49.2)	
**Bed net use**				0.031
0	8 (3.2)	5 (62.5)	3 (37.5)	
1	24 (9.7)	17 (70.8)	7 (29.2)	
2	28 (11.3)	10 (35.7)	18 (64.3)	
3	188 (75.8)	80 (42.6)	108 (57.4)	
**Time to travel to clinic/hospital**				0.078
Less than 1 hour	203 (81.9)	97 (47.8)	106 (52.2)	
About 2 hours	45 (18.1)	15 (33.3)	30 (66.7)	
**Wait for care**				0.218
Less than 1 hour	97 (39.1)	45 (46.4)	52 (53.6)	
About 2 hours	75 (30.2)	28 (37.3)	47 (62.7)	
More than 2 hours	76 (30.7)	39 (51.3)	37 (48.7)	
**Non mosquito causes**				0.078
No	111 (44.8)	57 (51.4)	54 (48.6)	
Yes	137 (55.2)	55 (40.2)	82 (59.8)	
**Malaria to typhoid**				
No	112 (45.2)			
Yes	136 (54.8)			

Abbreviations: SES: Socioeconomic status, n: Frequency, % : Percentage

P-values are calculated using chi-square test

Both the unadjusted and adjusted logistic regression analysis found a significant association between gender and belief about malaria to typhoid progression. The odds of belief in malaria to typhoid transmission were higher among females compared to males (crude OR, 95% CI) (1.87, 1.13 – 3.11), p= 0.015. Even after adjusting for potential confounders, the odds of belief in malaria to typhoid transmission were higher among females compared to males (adjusted OR, 95% CI) (2.15, 1.21 – 3.79), p=0.009 (Table 2).. There was no significant association between the outcome variable and education, SES, residence, age, land ownership, wait time for care, time to travel to the clinic, and non-mosquito causes.

**Table 2:** Factors associated with Malaria to Typhoid progression and study variables

	Crude OR (95% CI)	*p* value	Adjusted OR (95% CI)	*p* value
**Age**				
≤30 years	Reference		Reference	
31 - 55 years	1.05 (0.58, 1.89)	0.875	1.14 (0.60, 2.16)	0.692
>55 years	0.88 (0.44, 1.80)	0.733	1.49 (0.63, 3.56)	0.365
**Gender**				
Male	Reference		Reference	
Female	1.87 (1.13, 3.11)*	0.015	2.15 (1.21, 3.79)**	0.009
**Education**				
None	Reference		Reference	
Primary	2.09 (0.87, 5.02)	0.098	2.22 (0.78, 6.32)	0.136
Secondary	1.81 (0.73, 4.47)	0.200	2.81 (0.91, 8.70)	0.073
Tertiary	1.50 (0.34, 6.56)	0.590	3.08 (0.60, 15.97)	0.180
**SES**				
Low income	Reference		Reference	
Middle/high income	0.72 (0.43, 1.20)	0.210	0.72 (0.40, 1.27)	0.252
**Ruralurban**				
Non irrigated	Reference		Reference	
Irrigated	0.73 (0.42, 1.27)	0.267	1.11 (0.54, 2.29)	0.772
Urban	0.59 (0.28, 1.24)	0.164	0.51 (0.21, 1.26)	0.144
**Land in hectars**				
No land	Reference		Reference	
1-2 hectars	1.06 (0.59, 1.90)	0.840	1.14 (0.60, 2.18)	0.685
>2 hectars	0.76 (0.40, 1.44)	0.401	0.82 (0.40, 1.66)	0.576
**Bednet use**				
0	Reference		Reference	
1	0.69 (0.13, 3.69)	0.661	0.82 (0.13, 5.18)	0.835
2	3.00 (0.59, 15.26)	0.186	3.42 (0.59, 19.93)	0.171
3	2.25 (0.52, 9.69)	0.276	2.57 (0.51, 12.87)	0.252
**Time to travel to clinic/hospital**				
Less than 1 hour	Reference		Reference	
About 2 hours	1.83 (0.93, 3.61)	0.081	1.36 (0.61, 3.05)	0.449
**Wait for care**				
Less than 1 hour	Reference		Reference	
About 2 hours	1.45 (0.79, 2.69)	0.234	1.41 (0.70, 2.84)	0.341
More than 2 hours	0.82 (0.45, 1.50)	0.520	0.76 (0.38, 1.49)	0.416
**Non mosquito causes**				
No	Reference		Reference	
Yes	1.57 (0.95, 2.61)	0.079	1.50 (0.84, 2.67)	0.173

Abbreviations: SES: Socioeconomic status, OR: Odds ratio, CI: Confidence interval

* p<0.05

** p<0.01

## Discussion

The findings of the present study underscore malaria into typhoid progression is a widely held cultural belief system among the Kikuyu communities of Mwea with a significant of gender differences. However, this cultural belief system is not supported by clinical and scientific studies. This means people who believe on malaria into typhoid progression could potentially self-treat the illness with wrong medication that could have a negative health outcome. Self-treatment is the most common treatment seeking behavior in rural Kenya and countries in the region.^3^,^9^ Previous studies validate this finding by showing gender-based differences in the perception and experiences of illnesses.^3^,^7^,^22^ Earlier studies from Kenya and the region also support the results of this study by showing women of low socio-economic and educational status, with limited access to outside information (restricted to local cultural knowledge), are more likely to experience worse health outcomes and lack of reliable knowledge about diseases than women of higher socio-economic and educational status.^26^-^28^ These social and cultural factors potentially contribute to this gender disparity and the adherence of culturally ingrained health belief systems. Gender roles and expectations in many cultures lead to gender inequity in health. 29-31 Furthermore, the cultural and institutional inequalities limit women's access to other knowledge sources and restricts them to culturally prescribed knowledge.^32^ Lack of decision-making power, limited economic opportunity, and power inequalities also add to the overall gender disparity in health.^33^ In the current study, compared to females, males were more likely to have secondary or tertiary education and report higher self-ascribed SES , although these gender differences were not statistically significant.

The study findings that women have higher adherence to malaria to typhoid progression beliefs than men is also consistent with other studies that show there is a greater social expectation that women have to stick to culturally prescribed ideas and practices. 34,35 In many cultures around the world, women are expected to be loyal to their culture and follow it accordingly. Women who do not adhere to culturally prescribed ideas and beliefs are in many cases stigmatized or ostracized by both men and women in society.^36^ Women are not given the cultural and social space to question or do things outside of the cultural norm. The findings are important, confirming how gender inequality in health is woven in a web of oppressive cultural, and social systems that are associated with negative women health outcomes.^37^ Not only are traditional gender roles putting women at a greater health risk, but their inability to access information outside of their cultural space and questioning established cultural beliefs contributes to this elevated health risk.^33^ Access to education for women in Kenya and many other developing countries is limited compared to their male counterparts.^6^,^32^ The results of this study show how the extent and nature of indigenous cultural ideas help create a unique knowledge about the progression of one illness into another disease with significant gender differences.

This study is crucial to broaden our knowledge of the social, cultural, and structural forces that create gender health disparity in rural communities in the developing world. Belief systems are central to individual response to the treatment process. In most cases, culture labels the illness and defines the treatment. 8 An individual's compliance with treatment is a function of their cultural belief congruency to the prescribing medical system.^10^,^38^ Belief systems decide the course of treatment-seeking steps individuals should take. The study also provides vital contribution to the cultural context and the social meaning associated with malaria that has critical implications on gender-based health intervention strategies. This will play a major role in developing gender targeted health intervention and control in rural communities in the developing world. This study will also have contribution in the advancement of social theories and concepts to understand social process to gender health disparity.

However, this study is not without limitation. The study is based on self-reporting which might impact the validity of the study findings. Furthermore, this is not a randomized study; therefore, selection and social desirability related biases might impact the quality of the study results. Finally, the study did not do a lab test to rule out co-confection of malaria and typhoid based on gender that has the potential to bias the study result.

## Conclusion

Cultural belief systems play an important role in diseases diagnosis and treatment in many developing countries. The current result from this study emphasizes the urgency for integration of cultural and indigenous beliefs in our response to diseases. Significant gender-based differences in malaria to typhoid progression perceptions in this study highlights the need for gender-focused infectious diseases intervention in Mwea and other communities in the region. Many factors could influence women's health inequalities that includes lack of appropriate knowledge about malaria and typhoid. Access to knowledge, education and resources is not gender neutral. In many societies in the world, especially in the developing world, women's access to knowledge, education, and resources is very limited which negatively impacts their overall health outcomes. The results from this study may benefit in devising appropriate infectious diseases prevention and control programs that specifically target women and other vulnerable populations among rural communities in the developing world.
